# Exercise-induced arrhythmogenic right ventricular cardiomyopathy: Reverse remodeling with detraining

**DOI:** 10.1016/j.hrcr.2022.06.003

**Published:** 2022-06-17

**Authors:** Douglas Darden, Melvin M. Scheinman, Kurt S. Hoffmayer

**Affiliations:** ∗ Division of Cardiology, Department of Medicine, University of California, San Diego, La Jolla, California; † Division of Cardiology, Department of Medicine, University of California San Francisco, San Francisco, California

**Keywords:** Exercise, Arrhythmogenic right ventricular cardiomyopathy, Ventricular arrhythmia, Athlete, Detraining

**Key Teaching Points**

• Exercise-induced arrhythmogenic right ventricular cardiomyopathy (ARVC) is an under-recognized clinical entity without genetic abnormalities that has overlapping features of ARVC and athlete’s heart.
• An invasive electrophysiology study with programmed ventricular stimulation and high-definition endocardial voltage mapping may aid in the risk stratification process in select patients, although routine use is cautioned.
• In this case, exercise detraining may have a role in reversing the exercise-induced ARVC phenotype.

## Introduction

Long-term exercise training leads to structural cardiac adaptations, collectively referred to as the “athlete’s heart.” While the ventricles both undergo dilation and eccentric hypertrophy, it has been shown that the right ventricle (RV) experiences disproportionate remodeling under intense sports activity.^1^ Occasionally, the remodeling that occurs in the athlete’s heart may resemble arrhythmogenic right ventricular cardiomyopathy (ARVC), a pathologic cardiomyopathy associated with sudden death. Although rare, familial ARVC accounts for nearly a quarter of sports-related sudden cardiac death.^2^ It is well known that exercise is both a trigger for arrhythmias and contributes to a more severe phenotype of familial ARVC, which is caused by an autosomal dominant mutation in desmosomes leading to fibrofatty infiltration. Recent evidence has suggested a form of cardiomyopathy that fails to meet criteria for physiological athletic remodeling and resembles ARVC in the absence of genetic abnormalities in those participating in long-term intense exercise, referred to as “exercise-induced ARVC.”^1^^,^^3^

We present a case of an athlete diagnosed with exercise-induced ARVC that underwent an invasive electrophysiology study (EPS) with endocardial high-definition voltage mapping for further risk stratification and ultimately had complete reverse remodeling by imaging with exercise detraining.

## Case report

**Figure 1:**
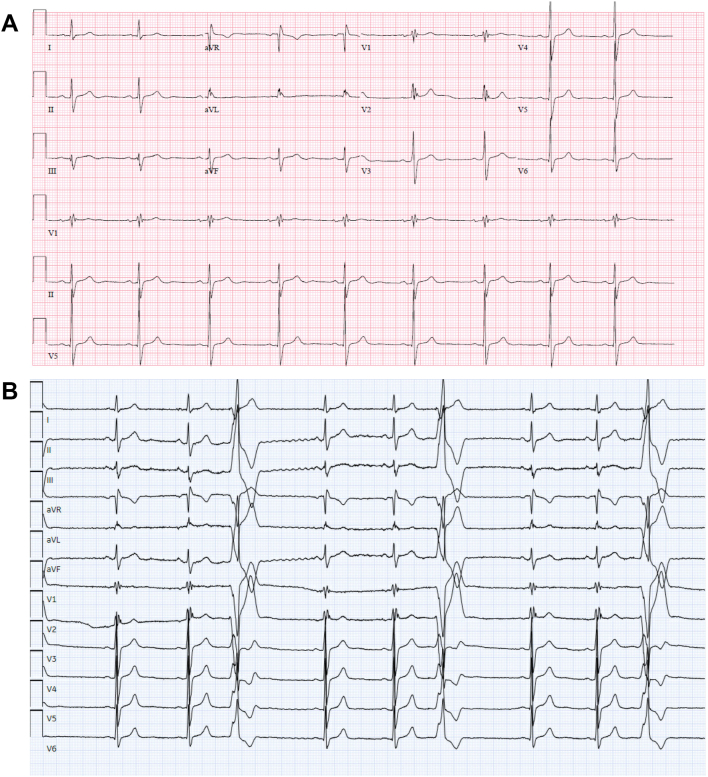
**A:** Baseline electrocardiogram prior to exercise detraining. **B:** Premature ventricular contraction with right ventricular outflow tract morphology in trigeminy pattern.

A 25-year-old athlete presented to his primary care physician with complaints of occasional palpitations that occur at rest. An electrocardiogram (ECG) demonstrated sinus bradycardia with an incomplete right bundle branch pattern (QRS 118 ms) with no T-wave inversions, along with a low-amplitude fractionation in the terminal phase of QRS complex, specifically with terminal activation delay in V_1_ of 60 ms (as measured from the nadir of the S wave to the end of depolarization), as shown in Figure 1A. Shortly after, he noted palpitations in office and a subsequent ECG demonstrated premature ventricular contractions (PVCs) in trigeminy pattern with a right ventricular outflow tract morphology (left bundle branch morphology, transition V_4_, inferior axis), shown in Figure 1B. A transthoracic echocardiogram revealed a normal left ventricle (LV) size and function and a moderately enlarged RV (RV diastolic area 34 cm^2^) with reduced function (tricuspid annular plane systolic excursion 12 mm).

The past medical history was unremarkable, with no illicit drug use, performance-enhancing drug use, or stimulant use. He reported no family history of cardiomyopathy, sudden cardiac death, or unexplained death in the family. He was a long-term endurance athlete participating in marathons, ultramarathons, and long-distance hiking, performing approximately 14 hours of endurance exercise per week for a decade.

**Figure 2:**
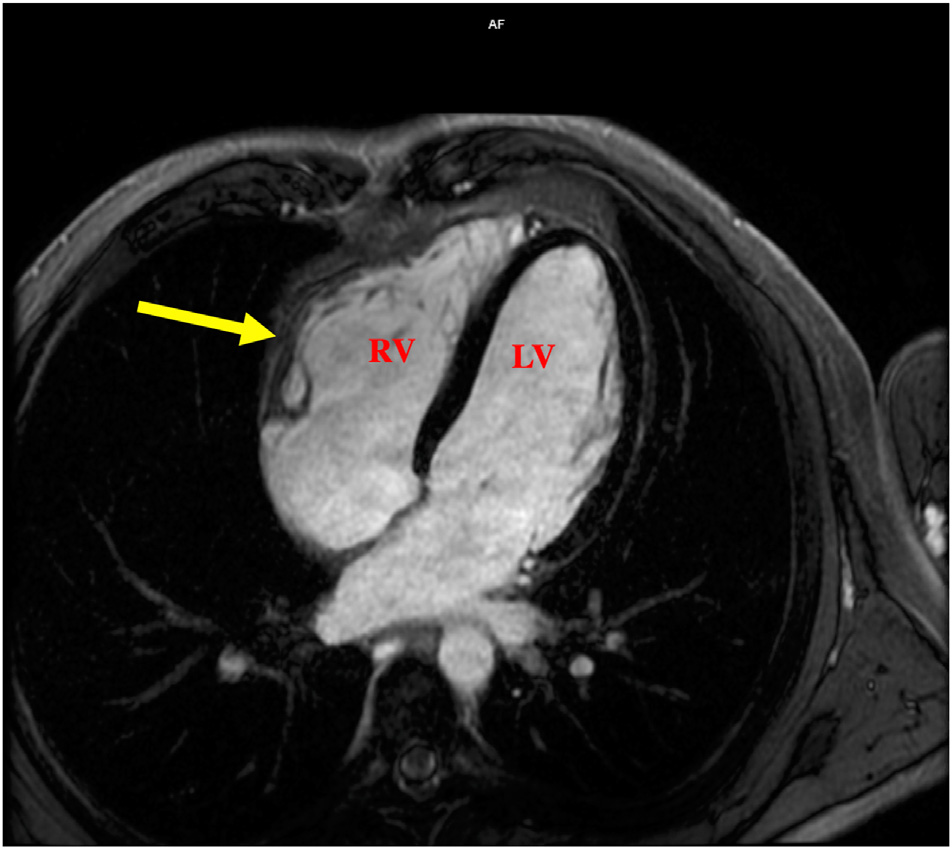
Enlarged right ventricle on cardiac magnetic resonance imaging. The yellow arrow points to the region of focal hypokinesis and mild aneurysm of the right ventricular basal to mid free wall.

A 14-day ECG monitor patch revealed predominantly sinus bradycardia with an average heart rate of 56 beats per minute with rare premature ventricular contractions (defined as <1%), although there was a total of 11,300 PVCs consisting of approximately 807 PVCs / 24 hours. No symptoms were noted. He underwent a submaximal exercise treadmill test with echocardiogram via the Bruce protocol, achieving 19 metabolic equivalents at maximal exertion. He was asymptomatic without wall motion abnormalities and had occasional PVCs during exercise and recovery (no 12-lead ECG available). Cardiac magnetic resonance imaging (CMR) demonstrated a mildly enlarged left ventricle (end-diastolic volume [EDV] 101.9 mL/m^2^) and mildly depressed LV ejection fraction (EF) of 49%, an enlarged RV (EDV 134.8 mL/m^2^) with mildly depressed global systolic function (EF 43%), and focal hypokinesis and mild aneurysm of the RV basal to mid free wall (Figure 2 and Supplemental Video 1A). There was no delayed gadolinium enhancement. Per the ARVC revised Task Force criteria, he met 1 major criterion (enlarged RV, depressed RVEF, and regional dyssynchrony) and 2 minor criteria (≥55 ms terminal activation duration in V_1_ without complete right bundle branch block and >500 PVCs / 24 hours), fulfilling a definite diagnosis of ARVC.^2^

He was referred to electrophysiology for concern of ARVC and was recommended to cease exercise, although he had already reduced his exercise to low intensity levels on his own volition. Importantly, genetic testing including a cardiomyopathy and arrhythmia genetic panel of more than 130 genes revealed no mutations, including 16 genes implicated in ARVC (*CTNNA3, DES, DSC2, DSG2, DSP, FLNC, JUP, LDB3, LMNA, PKP2, PLN, RYR2, SCN5A, TGFB3, TMEM43,* and *TTN*). Although he met criteria for ARVC, the lack of delayed gadolinium enhancement on CMR, no genetic mutation, and no sustained arrhythmias led to an unclear risk assessment for serious arrhythmia in this highly active patient. Furthermore, the patient had a desire to eventually resume high levels of endurance exercise and did not want to follow an exercise restriction indefinitely. After a shared decision-making discussion, an EPS with a ventricular tachycardia induction protocol and endocardial voltage mapping for further risk stratification was planned. If he had inducible monomorphic ventricular tachycardia, he was willing to continue restricting exercise and undergo subcutaneous implantable cardiac defibrillator implant (preferred over transvenous by patient).

**Figure 3:**
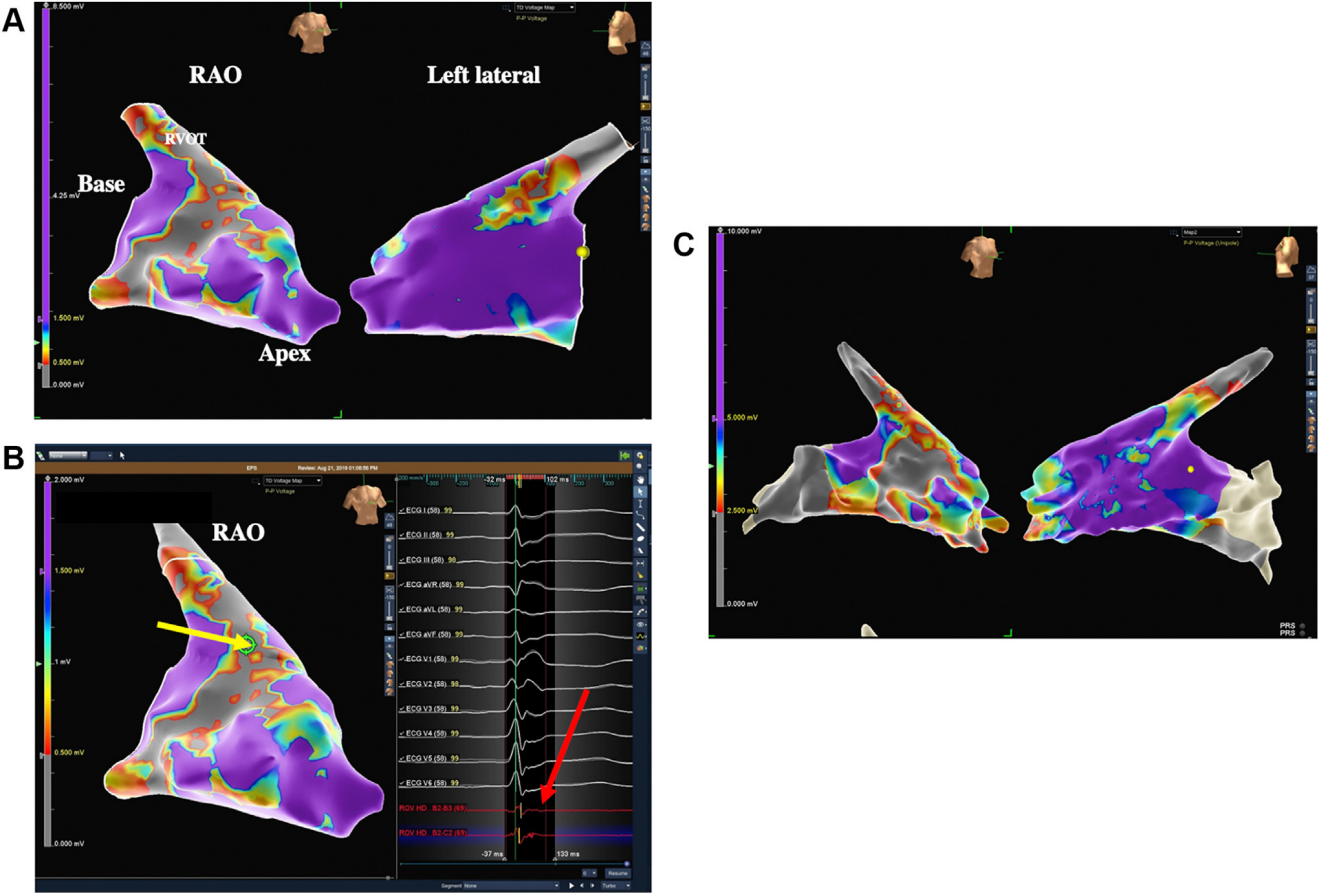
**A:** Endocardial voltage mapping of the right ventricle. **B:** Fractionated intracardiac signal (*red arrow*) shown obtained from basal aspect of right ventricular free wall just prior to the right ventricular outflow tract (*yellow arrow*). **C:** Unipolar right ventricular endocardial voltage maps. RAO = right anterior oblique; RVOT = right ventricular outflow tract.

A ventricular tachycardia induction protocol was performed with ventricular extrastimuli from the RV apex and RV outflow tract with single, double, and triple extrastimulus pacing with a drive train of 600 ms and 400 ms. The protocol was repeated while on high-dose isoproterenol at 20 mcg/min with no inducible ventricular arrhythmias, with tightest interval at 400 ms/220 ms/220 ms/220 ms. RV endocardial voltage was subsequently obtained using the EnSite Precision Navigation System and the Advisor High Density Grid, Sensor Enabled (Abbott Inc, Abbott Park, IL) mapping catheter, revealing endocardial bipolar low voltage (defined as <0.5 mV) in the basal lateral RV (Figure 3A) with fractionated intracardiac signals (Figure 3B). A similar distribution of unipolar low voltage (defined as <2.5 mV) was also observed, suggesting presence of epicardial scar (Figure 3C). These areas corresponded to the focal hypokinesis and mild aneurysm observed on the CMR. Additionally, a right heart catheterization was performed, showing normal filling pressures and no evidence of left-to-right shunt. He declined an invasive endomyocardial biopsy.

The definite diagnosis of ARVC as per Task Force criteria without genetic abnormality combined with a high lifelong dose of endurance activity led to the diagnosis of “exercise-induced ARVC.” To assess the possible relationship between exercise and ARVC, the patient agreed to continue a 6-month period of detraining. At 6 months, a resting 12-lead ECG was largely unchanged, with slight narrowing of the QRS duration (110 ms). However, repeat CMR demonstrated marked improvement, with now borderline RV enlargement and normal systolic function (EDV 94 mL/m^2^, RVEF 56%). LV size was also normal with preserved EF (61%). Palpitations ceased with no clinical arrhythmias. Repeat 14-day ECG monitor patch showed rare PVCs (<1%) and PVC burden decreased to approximately 80 PVCs / 24 hours. He was offered an implantable loop recorder for long-term monitoring; however, he deferred owing to lack of symptoms and was content with his exercise volume. At 1- and 2-year follow-up, he continued to refrain from endurance training and avidly participated in low-demanding cardiovascular activities, including yoga and golf, and had no clinical arrhythmias. Repeat CMR performed at both 1- and 2-year follow-up demonstrated complete normalization of RV size and function (Supplemental Video 1B).

## Discussion

This case provides insight into the under-recognized syndrome of “exercise-induced ARVC” with the use of an invasive EPS for ventricular arrhythmia stimulation protocol and high-definition voltage mapping to further aid in risk stratification. While there were no inducible ventricular arrhythmias, low bipolar and unipolar endocardial voltage were observed on invasive mapping that led to a continued exercise restriction recommendation that the patient followed. After 1 year of high-intensity endurance exercise restriction, the patient had no clinical arrhythmia, with normalization of biventricular size and function on CMR.

In 2003, Heidbüchel and colleagues^3^ first described exercise-induced ARVC in a series of 46 endurance athletes who presented with symptoms suggestive of ventricular arrhythmias. The vast majority had an RV arrhythmia origin and met Task Force Criteria for ARVC. Importantly, over a follow-up period of nearly 5 years, 18 (39%) experienced a major arrhythmic event, including 9 (20%) with cardiac arrest. Notably, induction of ventricular arrhythmia on invasive EPS was strongly associated with the major arrhythmic event in the follow-up period. However, it was still debated if exercise was simply accelerating the expression of familial ARVC in these athletes. Subsequent studies about a decade later demonstrated lower-than-expected desmosomal gene mutations in athletes with complex arrhythmias of RV origin, lending support to the notion that endurance exercise can lead to the phenotype of exercise-induced ARVC.^4^^,^^5^

It was hypothesized that sustained hemodynamic stress with inappropriate recovery may cause microscopic injury, weaken desmosomal integrity, and cause progressive RV dilation promoting arrhythmogenicity.^1^ These abnormal changes may be reversible, as evidenced in rat models subjected to endurance training.^6^ Still, once exercise-induced ARVC is diagnosed or considered clinically, uncertainty remains in risk-stratifying individuals for serious ventricular arrhythmias, particularly in our present case.

Although he had no clinical sustained arrhythmia, he still met a minor criterion for ARVC given >500 PVCs / 24 hours on his first monitor while he was already self-detraining. The role of an invasive EPS with programmed ventricular stimulation in risk stratification remains controversial, as conflicting data exist for its usefulness in the prediction of long-term arrhythmic outcomes.^3^^,^^7^, ^8^, ^9^ Supplementing the EPS with endocardial voltage mapping may provide added value in arrhythmic risk assessment in ARVC. In ARVC, fibrofatty replacement typically affects the epicardium and then extends inward into endocardium. Bipolar and unipolar voltage maps can serve as a surrogate for the endocardium and epicardium, respectively. Low-voltage areas, such as the subepicardial right ventricular outflow tract, may serve as an arrhythmogenic substrate and the extent of low endocardial voltage has been shown to strongly predict arrhythmic outcome.^8^^,^^10^ In addition to low endocardial bipolar and unipolar voltage in the RV basal to mid free wall in the current case, the fragmented electrograms within the low-voltage areas may also be a strong predictor of arrhythmic events, as described in patients with ARVC.^11^ It is important to note that CMR has been reported to be less sensitive than endocardial voltage mapping in identifying RV scar.^12^^,^^13^ The lack of delayed gadolinium enhancement in our patient may be explained by an early or mild form of ARVC with microscopic injury or thin layer of fibrosis that may not reach the threshold of CMR resolution. It also remains unclear if high-definition mapping may lead to greater discordance in CMR and voltage mapping findings. A histologic diagnosis via an endomyocardial biopsy could provide further diagnostic information; however, it was declined by our patient. Ultimately, the low-voltage areas observed on endocardial mapping supported the exercise restriction recommendation for our patient.

To the best of our knowledge, this case may be the first describing normalization of cardiac structure by imaging in a patient with exercise-induced ARVC, supporting the notion that exercise-induced ARVC may be acquired. However, several limitations are worth expanding on further. First, while we suggest detraining may have led to the normalization of cardiac structure and decrease in an already low baseline PVC burden, this should not be considered a causal relationship in the context of a single patient. Complete normalization should also not be presumed, since invasive voltage mapping was not repeated. Second, despite the thorough evaluation demonstrating no sustained ventricular arrhythmias, the relationship between exercise and arrhythmic risk could be underestimated, as he was already detraining prior to most of the evaluation, including the first continuous ECG monitor patch and invasive EPS (1 and 3 months of detraining, respectively). Third, he was also noted to have mildly enlarged LV with LVEF of 49% on initial magnetic resonance imaging. Although highly trained endurance may develop eccentric hypertrophy leading to mildly depressed LVEF, we cannot exclude LV abnormalities, as we did not perform invasive LV voltage mapping. It has been suggested that exercise-induced ARVC may exist on a spectrum involving biventricular and LV-dominant forms.^14^ Fourth, while we highlight the use of high-definition voltage mapping to supplement an invasive EPS using a ventricular arrhythmia stimulation protocol^3^ with the aim to further risk-stratify, we caution routine use of such strategy. Endocardial voltage mapping as a diagnostic and prognostic tool only has a class IIb recommendation as per the ARVC Internal Task Force Consensus Statement.^7^ The highly operator-dependent technique with chance of inaccurate readings owing to suboptimal catheter contact, risk of invasive procedure, and the cost, particularly with high-definition mapping catheters, need to be strongly considered.^7^ Lastly, although the genetic panel including over 130 genes with 16 genes implicated in ARVC revealed no mutations, we cannot exclude an undiscovered gene mutation in this phenotype.

In the absence of robust literature detailing management strategies for exercise-induced ARVC, the shared decision process becomes the foundation to guide management strategies. Importantly, after the diagnosis was obtained, our patient followed the recommendation to continue detraining. To most athletes, sport is an identity that provides intrinsic satisfaction and enjoyment that is difficult to discontinue. Appreciating the importance of sport and providing suitable alternatives, such as low-demanding cardiovascular sports, proves vital in a successful shared decision-making process. Further prospective studies and close surveillance are warranted in those athletes with normalization of exercise-induced ARVC, especially in those who desire to return to high levels of exercise.

## Conclusion

Exercise-induced ARVC is under-recognized, with features overlapping athlete’s heart and ARVC that should be considered among athletes presenting with symptoms concerning for arrhythmia in the absence of genetic abnormalities. While an invasive EPS with programmed ventricular stimulation and endocardial voltage mapping may aid in the arrhythmic risk assessment in select cases, the procedural risk and lack of robust data should prohibit routine use. Lastly, this case suggests exercise detraining may be an effective strategy to aid in the diagnosis, although further studies are needed.

## Appendix Supplementary data

Supplemental Video A Four-chamber steady-state free precession cine image via cardiac magnetic resonance imaging showing enlarged right ventricle with reduced systolic function, along with focal hypokinesis and mild aneurysm of the right ventricular basal to mid free wall.

Supplemental Video B Repeat cardiac magnetic resonance imaging showing structural biventricular normalization following two years of exercise detraining.
